# Allergy to Lipid Transfer Protein or Hypersensitivity to Non-Steroidal Anti-Inflammatory Drugs?

**DOI:** 10.3390/ijms26135988

**Published:** 2025-06-22

**Authors:** Magdalena Rydzyńska, Kinga Lis, Zbigniew Bartuzi, Tomasz Rosada, Magdalena Grześk-Kaczyńska, Natalia Ukleja-Sokołowska

**Affiliations:** 1 Clinic of Allergology, Clinical Immunology and Internal Diseases, Jan Biziel University Hospital No. 2 in Bydgoszcz, Ujejskiego 75, 85-168 Bydgoszcz, Poland; magdalena_rydzynska@outlook.com (M.R.);; 2 Department of Allergology, Clinical Immunology and Internal Diseases, Ludwik Rydygier Collegium Medicum in Bydgoszcz, Nicolaus Copernicus University in Toruń, 87-100 Toruń, Poland; kinga.lis@cm.umk.pl (K.L.); zbartuzi@cm.umk.pl (Z.B.); medtom@op.pl (T.R.)

**Keywords:** allergy, anaphylaxis, food allergy, non-steroidal anti-inflammatory drugs (NSAIDs), lipid transfer protein (LTP), single NSAID-induced urticaria/angioedema or anaphylaxis (SNIUAA)

## Abstract

Non-steroidal anti-inflammatory drugs (NSAIDs) can cause hypersensitivity reactions and lead to anaphylactic shock. These drugs also act as cofactors in allergic reactions. Lipid transfer proteins (LTPs), found in plants, represent a unique group of allergens in which cofactors play a crucial role. This case report describes a 26-year-old female who developed anaphylactic symptoms after ingesting grapes and taking ketoprofen. The patient experienced swelling of the lips, tongue, and throat, as well as shortness of breath, dizziness, and loss of consciousness, after consuming grapes and taking ketoprofen. She had previously used ketoprofen and acetylsalicylic acid without issues but had developed urticaria on several occasions after consuming multi-ingredient dishes. Skin prick tests showed positive results for peanut and orange allergens. Further testing using the ALEX multiparametric test detected antibodies to several LTP allergens. Intradermal tests with ketoprofen yielded a positive result, although irritant reactions could not be ruled out. A provocation test with acetylsalicylic acid (ASA) showed no adverse reactions. Skin tests with ibuprofen were negative, and provocation tests confirmed its tolerance. A diagnosis of LTP allergy and selective ketoprofen allergy was made, with the recommendation to avoid ketoprofen and follow a diet excluding foods from the LTP group.

## 1. Introduction

According to the World Allergy Organization, drugs are the most common cause of anaphylaxis in adults [1].

Non-steroidal anti-inflammatory drugs (NSAIDs) can cause hypersensitivity symptoms and may lead to anaphylactic shock. Hypersensitivity reactions can be either allergic, involving immunological mechanisms, or non-allergic [2,3]. Differentiating between these two mechanisms is often challenging. These drugs are also considered significant cofactors in allergic reactions. They can induce food-dependent NSAID-induced anaphylaxis (FDNIA) following the ingestion of certain foods [3,4]

Lipid transfer proteins (LTPs), which are temperature- and digestion-resistant prolamins found in plants, represent a unique group of allergens in which cofactors play a crucial role in the course of allergic reactions [5,6].

This case report describes a patient who experienced an anaphylactic reaction after ingesting grapes and ketoprofen.

## 2. Case Report

### 2.1. Patient-Relevant Clinical Data

A 26-year-old female was admitted to the Allergology Clinic for the evaluation of a suspected food allergy. A year earlier, after consuming grapes, she experienced swelling of the lips, tongue, and throat; shortness of breath; dizziness; and loss of consciousness. One hour before the meal, she had taken ketoprofen for cervical spine pain. Her symptoms resolved after the emergency team administered epinephrine, a corticosteroid, and an antihistamine. Initially, in accordance with hospital procedures, she was monitored in the hospital emergency department, but she refused full hospitalization in a dedicated hospital clinic.

Non-steroidal anti-inflammatory drugs (including ketoprofen and acetylsalicylic acid) had been taken by the patient many times before, without any symptoms of intolerance. However, the patient reported several episodes of urticaria after eating multi-ingredient meals and a single episode of urticarial blisters with accompanying swelling of the lips and eyelids and rhinitis, which occurred after eating a celery and pineapple salad.

The patient’s clinical history did not indicate any chronic diseases. In her family history, the woman reported psoriasis in her mother and rheumatoid arthritis in her grandmother. Her son was diagnosed with an allergy to egg whites and an inhalant allergy to grass pollen.

### 2.2. Standard Diagnostic Laboratory Test Results

The results of basic laboratory tests did not show any deviations from the norm (Table 1). There were no abnormalities in blood coagulation parameters (Table 1). The results of immunochemical tests showed vitamin D deficiency and the presence of antithyroid antibodies (Table 1).

As part of the allergy diagnostics, the ALEX multiparameter test (Allergy Xplorer, MDX Wienna) was used. The total concentration of immunoglobulin E (tIgE) in the patient’s serum was 1245 kU/L. Specific (sIgE) IgE was detected for the allergen components of several plant allergens, both of food and airborne origin, as well as for food allergens of animal origin and allergens of the common wasp venom. Specific IgE for the remaining allergens represented in the ALEX test was negative. A detailed list of positive ALEX test results is presented in Table 2.

### 2.3. Skin Prick Test (SPT) and Intradermal Test (IT) Results

As part of the standard allergological diagnostics, the patient underwent skin prick tests (SPTs) with a basic series of inhalant and food allergens. The components of both series and the results of the skin prick tests with these allergens are presented in Table 3.

Intradermal tests with ketoprofen were performed. Ketoprofen (Ketonal, 50 mg/mL, injection solution, SANDOZ GmbH, Basel, Switzerland) was used in two dilutions, 1:100 and 1:10 (Ketonal/NaCl 0.9%). A positive result occurred at a 1:10 dilution of the solution. No standardized concentrations have been established to distinguish irritant from allergic reactions for ketoprofen. The concentration used in this test is relatively high, which makes it impossible to definitively rule out an irritant reaction. The lack of standardized concentrations of irritants for ketoprofen is a significant limitation of this diagnostic approach.

In order to expand the diagnostics and simultaneously identify a safe drug from the group of non-steroidal analgesics and anti-inflammatory drugs, skin prick tests and intradermal tests with ibuprofen were performed, in which all results were negative.

### 2.4. Spirometry and Challenge Test Results

The patient’s history of anaphylaxis was a contraindication to the ketoprofen challenge test, so this challenge was waived. The patient was considered eligible for the acetylsalicylic acid (ASA) challenge test. Under intensive allergological monitoring, a challenge test was performed using a cumulative dose of 1000 mg ASA, which did not cause any adverse reactions.

Oral and intravenous ibuprofen provocation tests were also performed, using doses of 200 mg orally and intravenously. Ibuprofen at this dose was well tolerated regardless of the route of administration.

To extend the standard allergological diagnostics, a spirometric test with a histamine provocation test was performed, which revealed bronchial hypersensitivity in the patient. Further monitoring for bronchial asthma was recommended.

### 2.5. Non-Standard Experimental Laboratory Test Results

Due to the unclear cause of the patient’s clinical reactions, the complex nature of the observed symptoms, and the results of diagnostic tests, it was decided to perform non-standard laboratory tests. Non-standard in vitro tests are experimental studies that are of a research and development nature and are not diagnostic tests.

The secretion of interleukins (IL-1β, IL-5, IL-8, IL-18) by whole blood cells after stimulation with selected allergens was assessed. The allergens for stimulation were selected based on data from the interview and the results of previous diagnostic tests performed on the patient. Single allergens and their combinations were used for stimulation.

**Figure 1:**
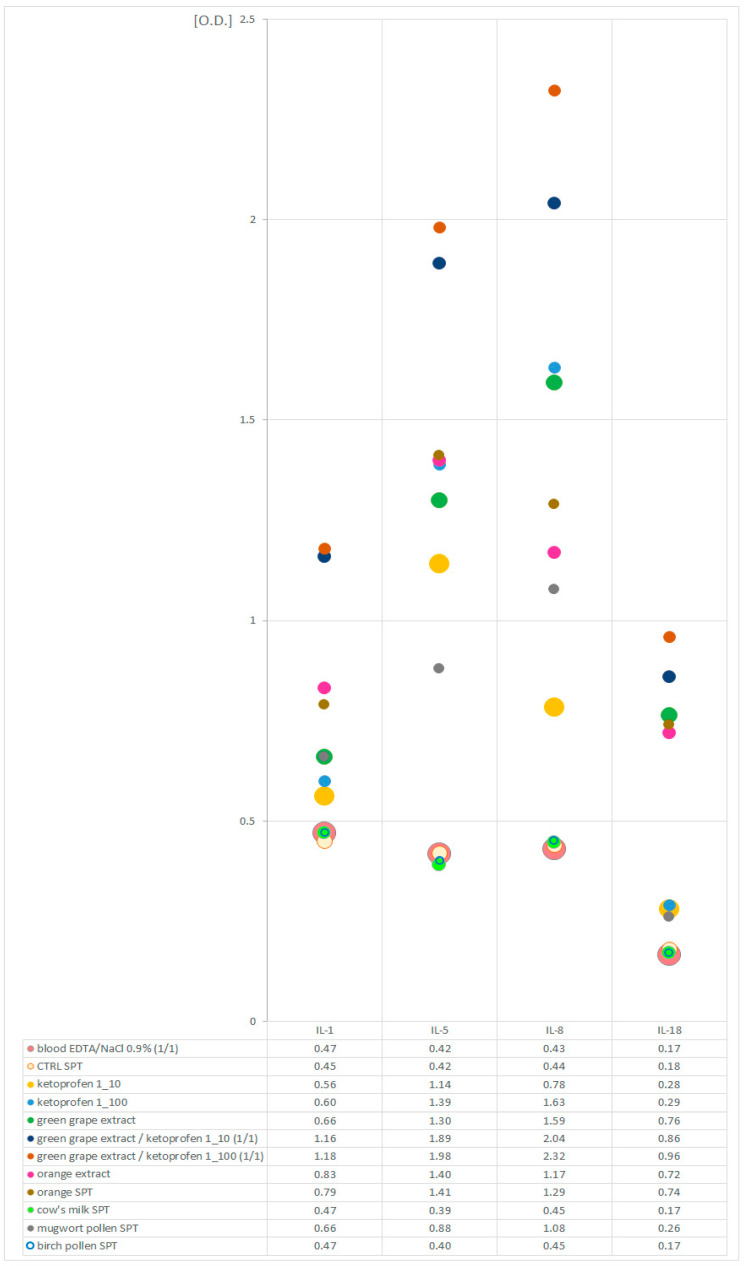
Levels of interleukins (IL-1β, IL-5, IL-8, IL-18) in allergen-stimulated EDTA blood samples and EDTA blood samples diluted with NaCl (0.9%) and negative control solution for skin tests (CTRL SPT).

The levels of cytokines in the sample diluted with physiological saline and in the sample diluted with a commercial negative control for skin prick tests (CTRL) were comparable. In blood samples stimulated with allergens selected as potentially sensitizing for the patient (green grape, orange, mugwort pollen, ketoprofen), an increase in cytokine secretion was observed in relation to the sample diluted with NaCl (0.9%) or to the sample diluted with the CTRL solution, respectively. This effect was amplified if the blood was simultaneously stimulated with a food allergen (grape) and ketoprofen. Stimulation with allergens potentially harmless to the patient (cow’s milk, birch pollen) did not cause increased cytokine secretion. The results of the experimental tests are presented in Figure 1.

### 2.6. Final Diagnosis and Recommendations for the Patient

The patient was discharged from the allergy clinic in good general condition. Based on the data from the interview, the general clinical picture, and the results of additional tests, an allergy to LTP was diagnosed. The patient was also recommended a diet excluding foods from the LTP group that had previously caused allergic reactions. The patient was additionally advised to avoid eating fruits and vegetables in combination with factors coexisting with the allergy. It was recommended to peel fruits and vegetables before eating. The patient was provided with detailed information on the sources and characteristics of LTP.

A selective allergy to ketoprofen was also diagnosed, and it was recommended to strictly avoid this drug. ASA and ibuprofen were considered safe alternatives. However, based on the results of the standard and non-standard tests, it could be assumed that hypersensitivity to ketoprofen was both a direct factor triggering clinical reactions in the patient and a coexisting factor in anaphylaxis in combination with the allergenic food.

Because of the risk of recurrent acute anaphylactic reactions, the patient was provided with epinephrine, a corticosteroid, and an antihistamine. She received comprehensive training in the management of anaphylaxis, including proper medication administration, and was instructed to carry an emergency kit with her at all times.

## 3. Discussion

The presented case highlights the coexistence of a food allergy and hypersensitivity to NSAIDs. It underscores the diagnostic challenges associated with the potential overlap between these two conditions. Additionally, it emphasizes the risks posed by NSAIDs acting as cofactors in allergic reactions.

### 3.1. Drug Hypersensitivity

Adverse drug reactions pose a significant threat to patient safety. Among these reactions, drug hypersensitivity is particularly noteworthy due to its acute and unpredictable nature. Drug hypersensitivity may result from both the active ingredient of a medication and the excipients contained within it. These reactions are dose-independent, unpredictable, and occur even when a drug is administered at doses typically tolerated by healthy individuals. Hypersensitivity can be classified as either allergic, involving immunological mechanisms, or non-allergic [7].

The mechanisms underlying allergic reactions include the formation of an allergen through the combination of a drug with a protein carrier in the body, the direct interaction of a drug with receptors on effector cells, or structural changes in antigen-binding receptors induced by the drug [8].

In contrast, non-allergic reactions may result from the non-specific stimulation of effector cells to secrete mediators, the inhibition of cyclooxygenase enzymes, the accumulation of bradykinin, the activation of the complement system, or the modulation of receptors [3,8,9].

The risk of hypersensitivity reactions depends on various factors, including the molecular weight of the drug, the duration of exposure, and the route of administration. Individual risk factors, such as gender, age, atopy, comorbidities, coexisting infections, and genetic predispositions, also play a crucial role in determining susceptibility.

Reactions are categorized based on their time of onset. Immediate reactions, which manifest within one hour of drug administration, typically include symptoms such as urticaria, angioedema, rhinitis, conjunctivitis, gastrointestinal disturbances, bronchospasm, and anaphylaxis. Non-immediate reactions, on the other hand, develop more than 24 h after drug administration and may present as rash, erythema, or inflammation of the internal organs, such as the kidneys, liver, or pulmonary alveoli, as well as hematologic abnormalities such as anemia, thrombocytopenia, and leukopenia [8,10].

The diagnostic approach for hypersensitivity reactions depends on their timing. Immediate reactions are investigated through skin tests and intradermal tests with immediate readings. In contrast, non-immediate reactions are diagnosed using patch tests and intradermal tests with delayed readings. The gold standard for the confirmation of hypersensitivity remains the drug challenge test. Non-steroidal anti-inflammatory drugs (NSAIDs) and antibiotics are among the most common triggers of drug hypersensitivity [2,8,9].

### 3.2. Non-Steroidal Anti-Inflammatory Drugs

Non-steroidal anti-inflammatory drugs (NSAIDs) are a widely used group of medications. They are available over the counter and are employed for their antipyretic, anti-inflammatory, and analgesic properties, making them suitable for a variety of conditions. Based on their chemical structures, NSAIDs can be categorized into several groups, including derivatives of acetylsalicylic acid, acetic acid, enolic acid, fenamic acid, pyrazolone, and alkanones. The primary mechanism of NSAIDs is the inhibition of the cyclooxygenase (COX) enzyme. These drugs are further classified into those that inhibit cyclooxygenase-1 (COX-1), which can be divided into strong and weak inhibitors, and those that selectively inhibit cyclooxygenase-2 (COX-2) [3].

The patient described in this case study took a drug from the NSAID group—specifically, a derivative of propionic acid, which is a strong COX-1 inhibitor.

### 3.3. Hypersensitivity to NSAIDs

Non-allergic hypersensitivity reactions to NSAIDs result from the cross-inhibition of cyclooxygenase-1 (COX-1) by various NSAID agents. COX-1 inhibition leads to decreased synthesis of prostaglandin E2, which, in turn, results in increased leukotriene synthesis and mast cell activation. This reaction predominantly involves strong COX-1 inhibitors. Disorders induced by cross-hypersensitivity include respiratory diseases exacerbated by NSAIDs, skin diseases exacerbated by NSAIDs, and NSAID-induced urticaria or angioedema [3,10].

In contrast, allergic reactions to NSAIDs depend on immunological mechanisms. Immediate allergic hypersensitivity reactions mediated by IgE include urticaria, angioedema, or anaphylaxis induced by a single NSAID (SNIUAA). Delayed hypersensitivity reactions induced by a single NSAID can also occur. Patients with such reactions generally tolerate NSAIDs with different chemical structures well [3,10,11].

According to the classification proposed by Romano et al., two additional phenotypes of NSAID-related hypersensitivity reactions are distinguished: NSAID-exacerbated food allergy (NEFA), which is a food allergy aggravated by NSAIDs, and NSAID-induced food allergy (NIFA), which is an anaphylactic reaction occurring only upon simultaneous exposure to NSAIDs and a specific food allergen. In a study conducted by Romano et al., involving 440 patients suspected of NSAID hypersensitivity who underwent skin prick tests, specific IgE measurements, and provocation tests with both drugs and foods, NSAID hypersensitivity was excluded in 162 patients, while, among 75 patients with a confirmed food allergy, nine were classified as NEFA cases and 66 as NIFA [12].

In the presented case, the patient experienced anaphylaxis triggered by ketoprofen consumption, although she reported prior tolerance to acetylsalicylic acid (ASA), another strong COX-1 inhibitor.

The diagnosis of NSAID hypersensitivity involves a comprehensive patient interview, which should include detailed information about the symptoms; the name, dose, and route of administration of the medication; and the tolerance of other NSAIDs both before and after the acute allergic reaction. This information helps to hypothesize the mechanism of the reaction. SNIUAA can be diagnosed using skin tests with the suspected drug [10].

To identify non-allergic cross-reactions, an oral provocation test is required. This test can be performed with the suspected drug to confirm the diagnosis, although this approach carries a risk of inducing an acute anaphylactic reaction. Alternatively, the test can be conducted with another NSAID—typically ASA—to diagnose cross-reactions, or with a likely tolerated NSAID [13].

To date, there is no standardization for skin and intradermal testing with NSAIDs. A universally established, non-irritant concentration of ketoprofen for use in such diagnostics has not been defined. According to the ENDA/EAACI Drug Allergy Interest Group position paper, the non-irritant concentrations of coxibs, pyrazolones, and other NSAIDs, such as ASA, ibuprofen, naproxen, indomethacin, diclofenac, meloxicam, and nimesulide, recommended for intradermal testing is 0.1 mg/L. However, these recommendations are based on limited case reports or case series [14]. For most drugs, there is a clear need for multicenter studies to establish standardized concentrations for skin and intradermal testing.

The diagnostic algorithm proposed by Doña et al. emphasizes the need for a thorough analysis of the patient’s medical history to avoid overlooking NEFA/NIFA phenotypes. In the subsequent diagnostic process, it recommends performing drug provocation tests, which are considered the gold standard in diagnosing drug hypersensitivity [15].

In this case, the patient underwent a skin test with ketoprofen, the drug taken prior to the anaphylactic reaction, albeit in a relatively high dose, which could also potentially provoke an irritant reaction. It is essential to consider the potential coexistence of factors, as the reaction involved both NSAID intake and the consumption of food from the LTP group. This scenario raised the possibility hypersensitivity to the drug might not have been present and that its role was limited to acting as a cofactor. Nevertheless, positive intradermal tests with ketoprofen raised the suspicion of ketoprofen sensitivity. Due to the history of anaphylaxis and the high risk of a severe allergic reaction, the patient was disqualified from an oral challenge with ketoprofen. The provocation test with ASA excluded cross-reactivity. Further diagnostic tests confirmed that ASA and ibuprofen were safe.

The absence of prior episodes of anaphylaxis of similar severity and the sudden onset of the incident may indicate a mechanism in which NSAIDs played a key role in initiating the immune response. On the other hand, the positive history of food allergies and confirmed hypersensitivity to LTP suggest that NSAIDs may have only exacerbated a preexisting allergy. Definitive differentiation between SNIUAA, NEFA, and NIFA remains challenging, and diagnostic reliability is limited by the lack of validated tests and the impossibility of performing provocation tests. Ultimately, the diagnosis of hypersensitivity to a single NSAID—ketoprofen—and allergy to LTP was established. The diagnosis of an LTP allergy and ketoprofen hypersensitivity was considered the most probable, safe, and practical from the perspective of further clinical management, especially regarding the prevention of future anaphylactic episodes.

### 3.4. NSAIDs as a Cofactor

NSAIDs are classified as cofactors in allergic reactions. By inhibiting the synthesis of prostaglandins, they reduce their protective effects on the gastrointestinal mucosa and disrupt the normal functioning of intestinal epithelial cells, thereby increasing the permeability of the gastrointestinal epithelial barrier [16,17]. Additional studies suggest that NSAIDs may induce the release of adenosine into the extracellular environment, trigger IgE-dependent reactions, or exert a direct effect by activating mast cells and basophils [18,19].

### 3.5. Lipid Transfer Proteins

Lipid transfer proteins (LTPs), which are molecules with a molecular mass of 6–11 kDa, belong to the prolamin superfamily. They are notable for their resistance to high temperatures and digestion. LTPs are found in fruits, vegetables, grains, nuts, tree and weed pollen, and latex. The highest concentrations of LTPs are present in the skin, the outer layers of fruits and vegetables, and in pits. Peach is the most common trigger of LTP-related allergies, with Pru p 3 serving as the primary sensitizer for other nsLTPs [6].

Some patients can tolerate fruits and vegetables after peeling them, although this does not fully eliminate the risk of an acute anaphylactic reaction. The presence of cofactors, such as physical exercise, menstruation, alcohol consumption, the use of NSAIDs, or infections, significantly increases the risk [20].

In the case described, the cofactor was an NSAID. Previously, the patient had consumed foods containing LTPs and experienced only mild symptoms. However, the concurrent intake of an LTP-containing food and an NSAID resulted in an anaphylactic reaction.

### 3.6. Non-Standard Experimental Laboratory Tests

Ex vivo tests involving the stimulation of biological material collected from a patient with an allergen in laboratory conditions have long been of interest to many researchers. This results, among other aspects, from the desire to make the most accurate diagnosis possible while minimizing the patient’s exposure to complications that may occur during in vivo challenge tests. Usually, stimulation is administered to cells isolated from the patient’s venous blood, their cultures, or cells remaining in whole blood as their natural environment. In recent years, the basophil activation test (BAT) has been introduced into the diagnostic procedure in allergies [21]. Another widely discussed ex vivo test considered in terms of minimally invasive allergy diagnostics is the mast cell activation test (MAT) [22]. Historically, these are not the only laboratory tests involving the stimulation of blood cells with allergens that have been attempted to be used in the diagnosis of allergic diseases. The Antigen Leukocyte Cellular Antibody Test (ALCAT) was initially promising, especially in relation to the diagnosis of allergies to inhalant allergens [23,24,25], as well as the Mediator Release Test (MRT) test in relation to the diagnosis of food allergies [26]. However, neither the ALCAT test nor the MRT has entered the canon of routine diagnostics, and they are currently perceived rather as methods that are not recommended for use in the diagnosis of allergies [27,28,29].

Nevertheless, and given these limitations, due to the unclear nature of the clinical symptoms occurring in the described patient, the ambiguous results of the standard tests performed, and the inability to perform safe provocation with an allergen suspected of causing an anaphylactic reaction involving a cofactor, we decided to perform non-standard laboratory experimental tests. Based on the assumptions of tests involving the ex vivo stimulation of blood cells with an allergen, we performed a series of allergen provocations of blood cells without their isolation and cell culture. The allergens were selected based on the results of previously performed routine tests. We challenged whole blood with allergens potentially allergenic for the patient individually and in combination with ketoprofen. The aim of these experiments was to confirm the hypersensitivity to the allergens (which were the source of LTP proteins) selected during earlier routine diagnostics and to verify the hypothesis about the role of ketoprofen as a cofactor in the allergic reaction. We assessed the responses of blood cells to the challenge by checking the levels of interleukins (IL-1β, IL-5, IL-8, and IL-18) in the plasma. It was assumed that an increase in the level of cytokines compared to their level in the plasma of unstimulated blood confirmed a positive response of the cells to the allergen challenge. The assumed cytokine profile was determined based on literature reports [30,31,32,33,34,35,36]. It was not characteristic of any specific group of white blood cells; therefore, it was not possible to assess the activation of a specific group of leukocytes using it. Therefore, we discuss the activation of leukocytes as such, without differentiating them.

The results of our experiments were consistent with the above assumptions. We observed the intensification of cytokine secretion in blood stimulated with allergens considered to be allergenic for the patient based on the results of earlier routine tests, with a lack of reaction to allergens to which the patient was not allergic. These observations seem to be confirmed by the earlier reports of other authors [30,31,32,33,34,35,36]. Moreover, the expression of the assessed cytokines was stronger when blood cells were challenged with a food allergen and ketoprofen simultaneously. This seems to confirm the important role of ketoprofen as a cofactor of the allergic reaction in the described patient. This part of the experiment is innovative. Similar allergen costimulation techniques have not been used before by other researchers.

We are aware of the limitations and weaknesses of this study. The main problem is that this study was performed with the participation of only one patient, which means that its results cannot be treated as evidence that the proposed test is effective in assessing clinical conditions associated with hypersensitivity to any allergens. This certainly requires studies involving a larger number of patients and a larger number of allergens. It would probably also be beneficial to expand the profile of cytokines assessed. We also note that the results of this experimental study could not be used in the diagnosis of the discussed patient. We based the clinical conclusions on routine diagnostics. Our non-standard experiment has scientific and cognitive value and may contribute to encouraging a larger group of researchers to conduct further research in this area.

## 4. Materials and Methods

### 4.1. Standard Diagnostic Laboratory Tests

Standard laboratory tests were performed using routine techniques and analytical systems used for this type of analysis in dedicated biological material collected in accordance with applicable rules. The procedures in force in the hospital and in accordance with the guidelines of the reagent manufacturers were followed. Basic diagnostic tests were performed (blood count with white blood cell differential, coagulation and fibrinolysis tests, basic biochemical tests, and vitamin D level). A broad-profile ALEX allergy test (Allergy Xplorer, MDX Wienna) was performed.

### 4.2. Skin Prick Tests, Intradermal Test, Challenge Tests, and Spirometry

Skin prick tests (SPTs), intradermal tests (ITs), challenge tests, and spirometry were performed in accordance with the requirements of internal procedures and using techniques and systems that are standard for this type of study.

The SPT was performed with two series of 10 allergens (food and inhalant) with commercial allergen solutions (Diater Laboratorio Farmacéutico, Madrid, Spain) intended for in vitro diagnostics. The list of allergens of both series is given in Table 3.

Intradermal tests were performed with a ketoprofen solution (Ketonal, 50 mg/mL, injection solution, SANDOZ GmbH, Basel, Switzerland) in two dilutions, 1:100 and 1:10, in physiological saline (NaCl 0.9%).

### 4.3. Non-Standard Laboratory Assays

Non-standard, experimental laboratory tests consisted of the stimulation of whole blood cells with allergens. Non-isolated blood cells were challenged with allergens alone and in combination with ketoprofen (as a potential cofactor of hypersensitivity reactions). The assessment of the effectiveness of allergen stimulation was based on the measurement of interleukin secretion (IL-1β, IL-5, IL-8, IL-18) before and after the ex vivo cell challenge process. The allergens for stimulation were selected based on the patient’s medical history and the results of the ALEX test and skin tests.

For stimulation, allergens were selected for which test results (in vitro or skin) were positive, i.e., food allergens (grape, orange), an inhalant allergen (mugwort pollen), and ketoprofen (in two dilutions), as well as allergens for which the results of previous tests were negative, i.e., a food allergen (cow’s milk) and an inhalant allergen (birch pollen). The experiment was performed using natural extracts (green grape, orange) and/or commercial extracts (orange, milk, mugwort pollen, birch pollen)—the same ones used in the skin tests (Diater Laboratorio Farmacéutico, Madrid, Spain). Natural extracts were prepared in-house on the basis of physiological saline.

To exclude the influence of the physiological saline or diluent used in the skin test extracts, the level of analyzed cytokines was also measured in blood diluted with 0.9% NaCl and in blood diluted with a solution designated as a negative control in the series of allergens used for skin tests. The levels of cytokine expression in these samples were used as a reference point for all samples stimulated with allergens.

The test was performed on whole blood collected with the addition of dipotassium edetate (K2EDTA). This is a sample typically used for blood count testing. Stimulation was performed in laboratory conditions immediately after blood collection from the patient. In order to stimulate blood cells, well-mixed whole blood and allergen solutions were mixed in equal volumes in clean, sterile test tubes. An appropriate proportion converter was used for ketoprofen solutions so that the target concentration was equivalent to the dilutions that were originally used in the intradermal tests. Samples prepared in this way were incubated at 37 °C for 24 h. Then, the samples were centrifuged (3000 g/15 min in 18–24 °C) to obtain plasma. Cytokines were measured in plasma using commercial ELISA tests (HumanIL-1B RD194559200R; Human IL-8 RD194558200R, BioVendor—Laboratorni medicina a.s. Karasek 1767/1 621 00 Brno Czech Republic; IL-5 SEA078Hu; IL-18 SEA064Hu; Cloud-Clone Corp. 23,603 W. Fernhurst Dr., Unit 2201, Katy, TX 77494, USA). All ELISA tests were performed according to the manufacturer’s instructions. Due to the use of atypical material, the determination of cytokine concentrations based on the calibration curves included in the ELISA kits used was abandoned, and the levels of interleukins were expressed using the extinction coefficient (O.D.) value. All results were corrected for the background value determined for NaCl 0.9%.

## 5. Summary

Allergy to LTPs is increasingly recognized as a significant problem, not only in Mediterranean countries. Cofactors of allergic reactions, such as alcohol consumption, physical exertion, or the use of NSAIDs, play a particularly critical role in this type of allergy. Patients often do not experience severe symptoms from exposure to LTPs alone, but symptoms can escalate in the presence of a cofactor. Hypersensitivity to NSAIDs is also a relatively common issue. Patients may exhibit symptoms in response to a single NSAID or across the entire drug class.

This case highlights the diagnostic challenges involved in distinguishing between hypersensitivity to NSAIDs, LTP allergies, and the role of NSAIDs as cofactors in LTP-related allergic reactions.

It cannot be excluded that, in the case of the described patient, future diagnostic advances, including the development of validated immunological tests, may allow the revision of the current diagnosis. The decision not to perform a provocation test due to the high risk, along with the limitations of the available testing methods, highlights the existing gaps in non-invasive drug allergy diagnostics. This case underscores the need for further research into methods that enable the precise differentiation of NSAID hypersensitivity reactions, especially regarding their roles as cofactors in food allergies.

## Figures and Tables

**Table 1:** Results of standard laboratory tests performed on the patient.

Parameter	Result	Unit	Reference Value
Blood count with white blood cell differential			
White Blood Cells (WBC)	5.80	G/L	3.9–10.2
Red Blood Cells (RBC)	4.31	T/L	4.0–5.0
Hemoglobin (Hb)	13.0	g/dL	12.0–16.0
Hematocrit (HCT)	38.0	%	37.0–47.0
Mean Corpuscular Volume (MCV)	90.5	fL	80.0–99.0
Mean Corpuscular Hemoglobin (MCH)	30.2	pg	27.0–33.5
Mean Corpuscular Hemoglobin Concentration (MCHC)	33.3	g/dL	31.0–37.0
Platelets (PLT)	252	G/L	130–400
Mean Platelet Volume (MPV)	11.8	fL	8.5–12.5
Neutrophils	3.77	G/L	1.5–1.7
Basophils	0.11	G/L	0.02–0.55
Eosinophils	0.03	G/L	0.00–0.20
Lymphocytes	1.41	G/L	1.1–4.5
Monocytes	0.46	G/L	0.1–1.0
Inflammation parameters			
Erythrocyte Sedimentation Rate (ESR)	8	mm/h	<11
C-Reactive Protein (CRP)	<0.6	mg/L	<5.0
Basic biochemistry			
Sodium	137	mmol/L	135–145
Potassium	4.44	mmol/L	3.5–5.0
Creatinine	0.71	mg/dL	0.51–0.95
eGFR (Estimated Glomerular Filtration Rate; according to CKD-EPI)	118	mL/min	>90
Urea	30	mg/dL	17–48
Glucose (serum)	81	mg/dL	70–99 (fasting)
Immunochemical tests			
Thyrotropin (TSH; 3rd generation test)	0.627	mU/L	0.270–4.200
25-(OH) vitamin D	10.83	ng/mL	Deficit: <20 Low: 20–30 Optimal: 30–50 High: 50–100
Anti-Peroxidase Antibodies (anti-TPO)	107.2	IU/mL	<34
Anti-Thyroglobulin Antibodies (anti-TG)	85.6	IU/mL	<115
Coagulation and fibrinolysis parameters			
Prothrombin Time (PT)	11.2	seconds	9.6–14.3
International Normalized Ratio (INR)	0.95		0.8–1.2
Activated Partial Thromboplastin Time (APTT)	33.0	seconds	23–37

**Table 2:** Positive results for specific IgE detected in ALEX test.

Source	Allergen	Extract (E)/Molecule (M)	Protein Family	sIgE [kUA/L]
London plane (*Platanus acerifolia*)	Pla a 3	M	nsLTP	2.7
Hemp (*Cannabis sativa*)	Can s 3	M	nsLTP	7.48
Hemp (*Cannabis sativa*)	Can s	E	-	1.1
Common mugwort (*Artemisia vulgaris*)	Art v 3	M	nsLTP	2.23
Peanut (*Arachis hypogaea*)	Ara h 9	M	nsLTP	1.31
Maize (*Zea mays*)	Zea m 14	M	nsLTP	7.73
Maize (*Zea mays*)	Zea m	E	-	5.23
Strawberry (*Fragaria × ananassa*)	Fra a 1 + Fra a 3	M + M	PR-10 + nsLTP	1.98
Apple (*Malus domestica*)	Mal d 3	M	nsLTP	1.79
Peach (*Prunus persica*)	Pru p 3	M	nsLTP	1.26
Grape (*Vitis vinifera*)	Vit v 1	M	nsLTP	0.68
Celery (*Apium graveolens*)	Api g 2	M	nsLTP	2.12
Tomato (*Solanum lycopersicum*)	Sola l 6	M	nsLTP	0.36
Common walnut (*Juglans regia*)	Jug r 3	M	nsLTP	10.37
Blue mussel (*Mytilus edulis*)	Myt e	E	nsLTP	0.38
Common wasp (*Vespula vulgaris*) venom	Ves v 5	M	Antigen 5	0.55

**Table 3:** Composition of allergen series and results of skin prick tests.

Inhalant Allergens		Food Allergens	
Allergen	SPT result	Allergen	SPT result
Hazel (pollen)	Negative	Peanut	Positive
Silver birch (pollen)	Negative	Orange	Positive
Alder (pollen)	Negative	Cow milk (full extract)	Negative
Common mugwort (pollen)	Negative	Hen’s egg yolk	Negative
Common rye (pollen)	Negative	Hen’s egg white	Negative
Grass—mix (pollen)	Negative	Cow milk—casein	Negative
Cat dander	Negative	Cod	Negative
*Dermatophagoides farinae*	Negative	Shrimp	Negative
*Dermatophagoides pteronyssinus*	Negative	Hazelnut	Negative
*Alternaria alternata*	Negative	Wheat flour	Negative
*Cladosporium herbarum*	Negative	Soy flour	Negative

## Data Availability

Data are contained within the article.
